# Correlation between C-reactive protein levels and affecting factors for adiposity in apparently healthy Korean adolescents

**DOI:** 10.1186/1687-9856-2015-S1-P69

**Published:** 2015-04-28

**Authors:** Ki Eun Kim, Yong-Jae Lee, Yoon-Ho Shin, Hye-Jung Shin

**Affiliations:** 1 Department of Pediatris, CHA Gangnam Medical center, CHA University, Seoul, Korea; 2 Department of Family Medicine, Yongin Severance hospital, Yensei University, Geonggi, Korea; 3 Department of Pediatrics, National Medical Center, Seoul, Korea

## Aims

Recent studies have shown that C-reactive protein is not just an indicator of cardiovascular disease (CVD) incidence and mortality. Thus, early detection of a continous increase in CRP concentrations may be useful in predicting subsequent development of CVD or metabolic syndrome.

The objective of this study was to analyze high sensitivity C-reactive protein (hs-CRP) in apparently healthy young Korean adolescents and determine confounding factors for high hs-CRP in this population.

## Methods

We enrolled 197 middle school students (93 boys and 104 girls) who participated in a general health check-up at a tertiary hospital in Seoul. We measured height, weight, waist circumference and blood pressure and investigated hs-CRP concentrations, insulin levels, insulin resistance and lipid profiles. Hs-CRP levels were measured using the Behring BN II nephelometer (Dade Boering, Marburg, Germany) and log-transformed for analysis.

## Results

hs-CRP concentration was significantly higher in boys than in girls (P=0.012). Pearson’s correlation coefficients revealed a significant correlation between log- transformed hs-CRP and BMI (r=0.24, P=0.0008), waist circumference (r=0.24, P=0.0007), systolic (r=0.17, P=0.019) and diastolic blood pressure (r=0.23, P=0.014), and ALT (r=0.16, P=0.023). In stepwise multivariate linear regression analysis, sex (male gender), waist circumference, and diastolic blood pressure were positively and fasting serum HDL-cholesterol level was negatively associated with log-transformed hs-CRP.

## Conclusions

We found that there exists a gender difference in hs-CRP concentrations in apparently healthy adolescents and that log-transformed hs-CRP concentrations were positively associated with male, waist circumference and diastolic blood pressure and negatively associated with HDL-cholesterol level. The gender difference and the contibuting factors for hs-CRP found in these healthy adolescents suggests a possibly relevant pathophysiological mechanism involved in the increase of cardiovascular risk associated with childhood obesity.

